# Supplementary material for: Stabilizing the Exotic Carbonic Acid by Bisulfate Ion
Source: Molecules. 2021 Dec 21;27(1):8. doi: 10.3390/molecules27010008 (PMC8746525; doi:10.3390/molecules27010008)
Supplement: Supplementary file 1 [file molecules-27-00008-s001.zip › molecules-1473237-supplementary_corr.pdf]

Supplementary Materials

Stabilizing the Exotic Carbonic Acid by Bisulfate Ion

Huili Lu [1], Shi-Wei Liu [2], Mengyang Li [4], Baocai Xu [3,*], Li Zhao [3], Tao Yang, [4,*] and Gao-Lei Hou [4,*]

[1] Master Kong Beverage R&D Center, Shanghai 201103, China; luhuili@masterkong.com.cn
[2] China National Research Institute of Food & Fermentation Industries Co., Ltd., Beijing 100015, China; ff_nns@163.com
[3] School of Light Industry, Beijing Technology and Business University, Beijing 100048, China; zhaol@btbu.edu.cn
[4] MOE Key Laboratory for Non-Equilibrium Synthesis and Modulation of Condensed Matter, School of Physics, Xi'an Jiaotong University, Xi'an 710049, China; lmy0916@stu.xjtu.edu.cn
* Correspondence: chemglhou@gmail.com (G.-L.H.); taoyang1@xjtu.edu.cn (T.Y.); xubaoc@btbu.edu.cn (B.X.)

Table of Content

Figure S1. Three conformers of isolated carbonic acid molecule, bisulfate ion, and *cis-cis* sulfuric acid molecule.

Figure S2. Molecular orbitals of the optimized isomers of $[H_2CO_3{\cdot}HSO_4]^-$.

Figure S3. Intrinsic reaction coordinate (IRC) scan for the stepwise conversion of isomer **1** to isomer **4** via an intermediate state **in14**.

Figure S4. Animations showing the AIMD simulations for isomers **1** and **2a**.

Figure S5. Molecular graphs of $[H_2CO_3{\cdot}HSO_4]^-$.

Figure S6. Vibrational vectors for the selected vibrational modes of the isomers of $[H_2CO_3{\cdot}HSO_4]^-$.

Figure S7. Vibrational vectors for the imaginary vibrational modes of the transition states of $[H_2CO_3{\cdot}HSO_4]^-$.

Table S1. Charge distributions from natural population analysis for the different isomers of $[H_2CO_3{\cdot}HSO_4]^-$.

Table S2. Cartesian coordinates.

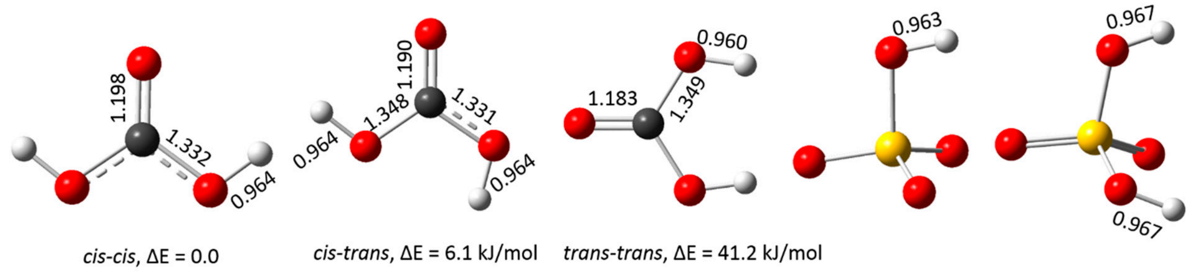

Figure S1. Three conformers of isolated carbonic acid molecule, bisulfate ion, and *cis-cis* sulfuric acid molecule at M06-2X/aug-cc-pVTZ level of theory. The bond lengths are shown in Å, and the relative stabilities of the three conformers of carbonic acid are obtained including zero-point energy corrections.

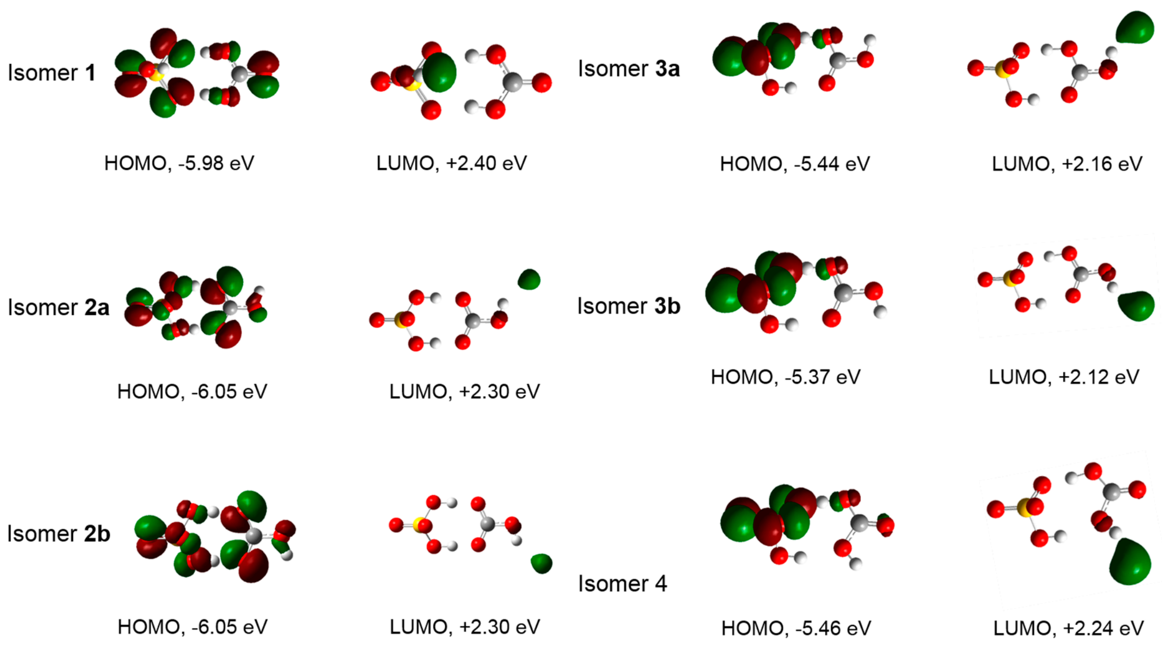

Figure S2. Highest occupied molecular orbitals (HOMOs) and lowest unoccupied molecular orbitals (LUMOs) of the optimized isomers of $[H_2CO_3{\cdot}HSO_4]^-$ (isovalue = 0.04). Energy levels of the MOs are provided.

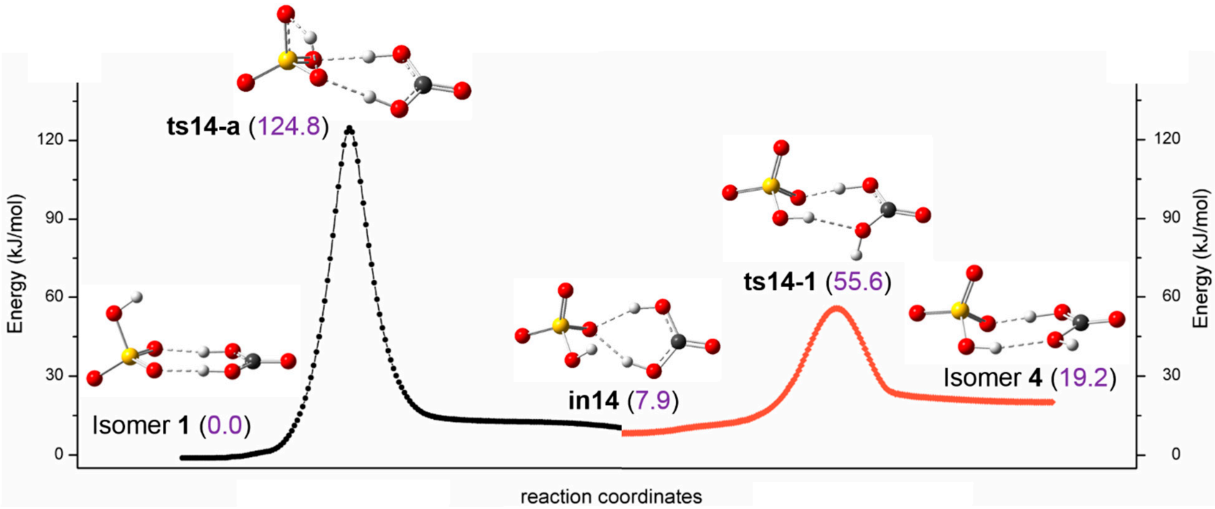

Figure S3. Intrinsic reaction coordinate (IRC) scan for the stepwise conversion of isomer **1** to isomer **4** via an intermediate state **in14**.

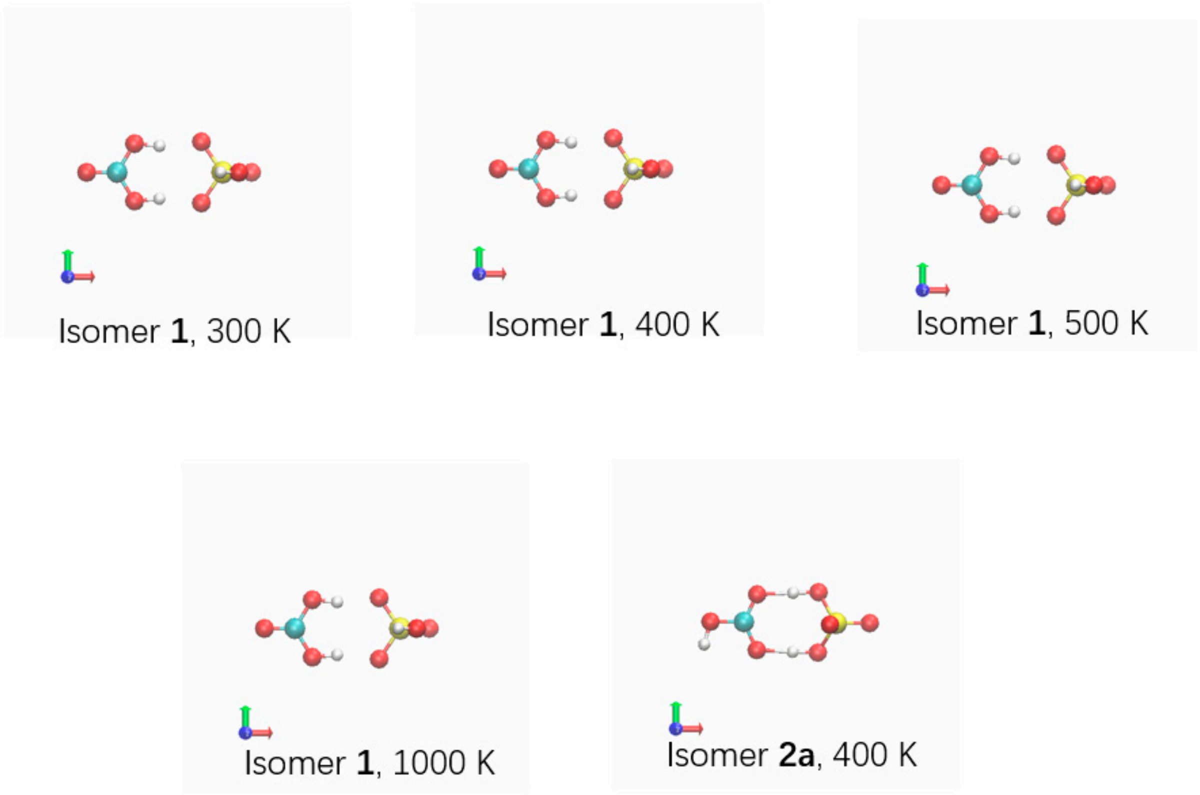

Figure S4. Animations showing the AIMD simulations for isomers **1** and **2a**. The total simulation time is 10 ps with a step of 1 fs (see in a separate .ppt file in display mode).

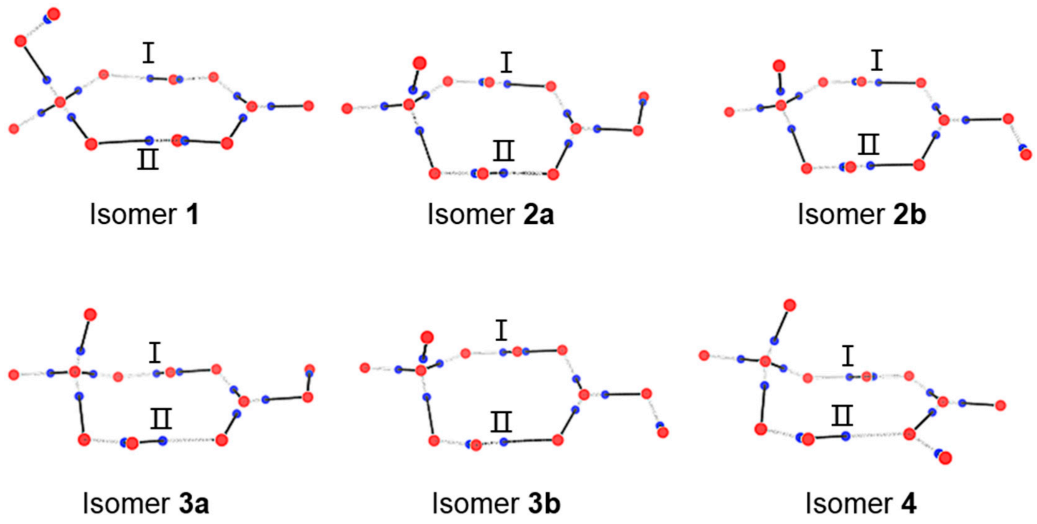

Figure S5. Molecular graphs of $[H_2CO_3{\cdot}HSO_4]^-$ showing the BCPs and the notation of hydrogen bonds I and II in each isomer.

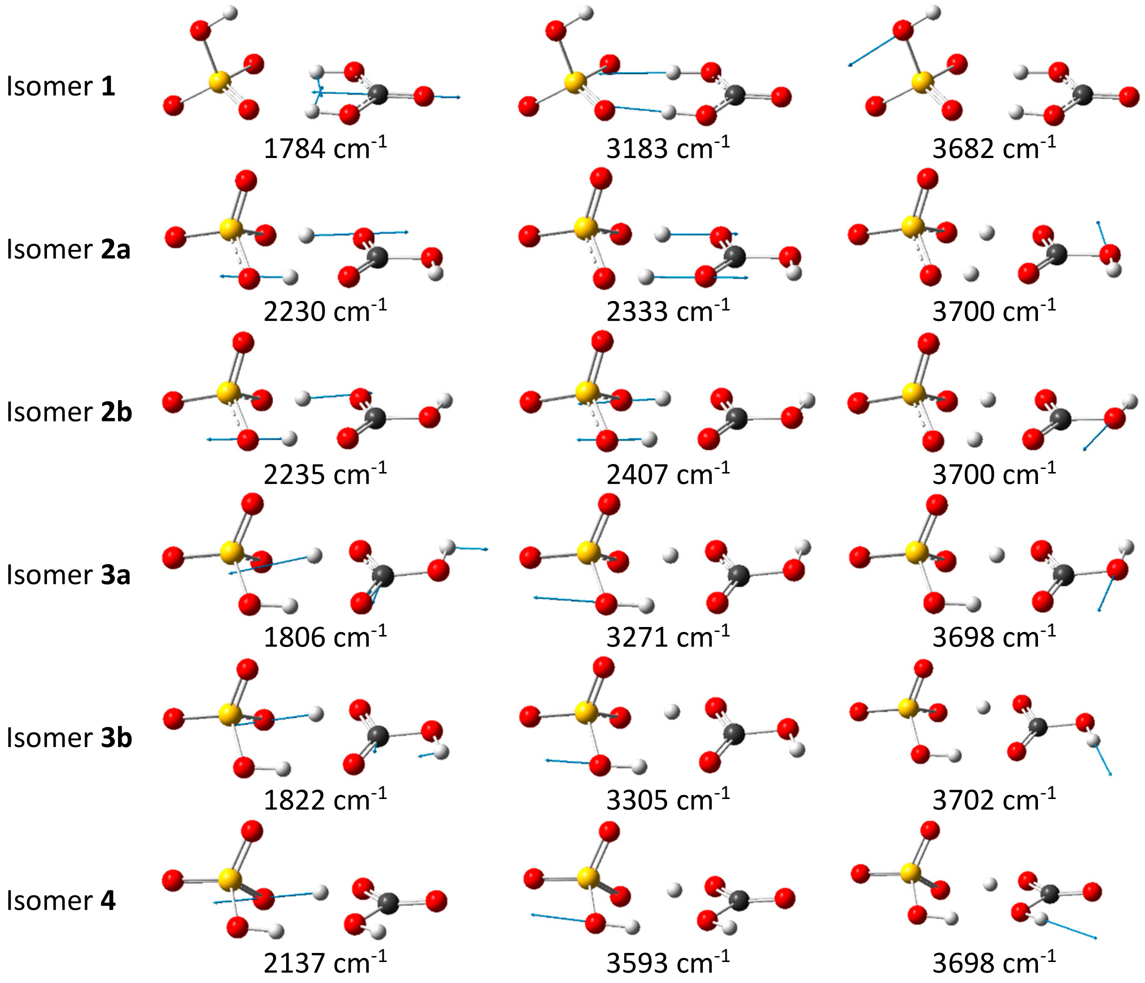

Figure S6. Vibrational vectors for the selected vibrational modes of the isomers of $[H_2CO_3{\cdot}HSO_4]^-$.

ts14-1

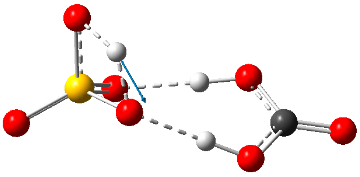

1705i cm^{-1}

ts14-2

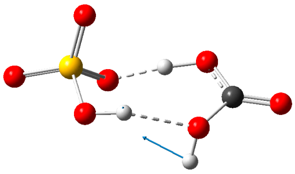

485i cm^{-1}

ts42b

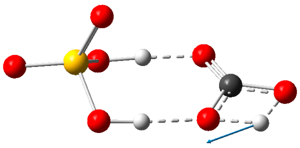

1748i cm^{-1}

ts2b3b

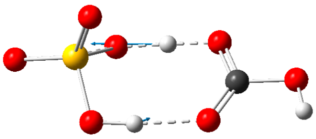

355i cm^{-1}

ts3b3a

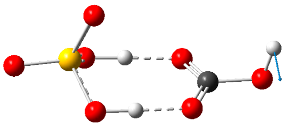

489i cm^{-1}

ts2a3a

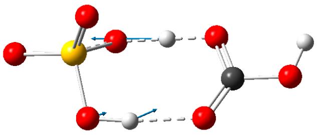

183 cm^{-1}

ts2b2a

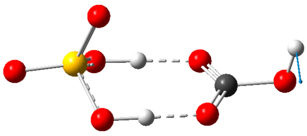

489i cm^{-1}

Figure S7. Vibrational vectors for the imaginary vibrational modes of the transition states of $[H_2CO_3{\cdot}HSO_4]^-$.

Table S1. Charge distributions from natural population analysis for the different isomers of $[H_2CO_3 \cdot HSO_4]^-$.

Isomers	HSO_4^-	H_2CO_3
Isomer **1**	-0.91	-0.09
Isomer **2a**	-0.24	-0.76
Isomer **2b**	-0.24	-0.76
Isomer **3a**	-0.85	-0.15
Isomer **3b**	-0.86	-0.14
Isomer **4**	-0.88	-0.12

Table S2. Cartesian coordinates for all the structures shown in Figures 1 and 2.

Isomer **1**
E(RM062X) = -964.851370409 A.U.

S	1.57786100	0.00005300	-0.12358600
O	2.73365800	0.00043400	-0.98156700
O	0.74379900	-1.21045400	-0.17115500
O	2.22691500	-0.00065500	1.36884900
H	1.49524100	-0.00053300	1.99640400
O	0.74383900	1.21064000	-0.16995900
H	-0.92379400	1.04698400	-0.11998400
C	-2.59805600	-0.00000600	-0.00298600
O	-1.90678600	1.13648500	-0.05017600
O	-3.79783300	-0.00003300	0.08409600
O	-1.90673200	-1.13646100	-0.05018300
H	-0.92376500	-1.04691200	-0.12037000

Isomer **2a**
E(RM062X) = -964.849443144 A.U.

S	1.54530600	-0.00988400	0.08629200
O	2.90742600	-0.00724300	-0.37552900
O	0.81334200	-1.19796100	-0.60857500
O	1.30181800	-0.06755100	1.50965700
H	-0.20686100	-1.17030900	-0.39387800
O	0.84164800	1.24630800	-0.50879800
H	-0.18629400	1.21681400	-0.32531900
C	-2.17038200	0.04166600	-0.04685500
O	-1.65173000	-1.09508400	-0.15175700
O	-1.63573700	1.15760300	-0.13082100
O	-3.51866900	0.05547300	0.18859200
H	-3.77422500	-0.87070900	0.23750200

Isomer **2b**
E(RM062X) = -964.849443254 A.U.

S	-1.54534500	-0.00995200	0.08624500
O	-2.90737900	-0.00742100	-0.37583500
O	-0.84185100	1.24661200	-0.50835900
O	-1.30213800	-0.06794500	1.50964600
H	0.18602400	1.21704100	-0.32502100
O	-0.81300100	-1.19763200	-0.60880400
H	0.20725100	-1.17003200	-0.39383200
C	2.17042800	0.04168700	-0.04678400
O	1.63570500	1.15757000	-0.13062500
O	1.65180900	-1.09511600	-0.15155800
O	3.51876900	0.05552500	0.18834300
H	3.77437000	-0.87064700	0.23717400

Isomer **3a**
E(RM062X) = -964.848187314 A.U.

S	1.54082000	-0.07417000	0.07768200
O	2.93307800	-0.13958500	-0.29609300
O	0.69395600	-0.99342600	-0.76897600
O	1.21874800	-0.22686800	1.48503500
H	-0.56058800	-1.02886600	-0.44499500
O	1.06545700	1.38803900	-0.36170200
H	0.08585800	1.41748900	-0.26607200
C	-2.18647200	0.08518400	-0.05531200
O	-1.65887400	-1.07957200	-0.21561700
O	-1.65658800	1.17460500	-0.14334100
O	-3.51100500	0.02620800	0.24043800
H	-3.73773100	-0.90820800	0.28207000

Isomer **3b**
E(RM062X) = -964.848570260 A.U.

S	1.54588600	-0.06060800	0.07858200
O	2.93952400	-0.12294900	-0.29263700
O	0.70896700	-1.02312300	-0.72139500
O	1.22889600	-0.14588900	1.49314800
H	-0.57287500	-1.07663900	-0.39760300
O	1.05413100	1.37877700	-0.42647800
H	0.07678600	1.39630000	-0.32417000
C	-2.19095900	0.00535600	-0.05507400
O	-1.65566200	-1.14559000	-0.18174000
O	-1.66942400	1.10835600	-0.16136700
O	-3.51652700	-0.07674300	0.22580500
H	-3.81156600	0.83521300	0.31222300

Isomer **4**
E(RM062X) = -964.843719843 A.U.

S	1.55221000	-0.08687500	0.08315700
O	2.95289700	-0.27230500	-0.21359400
O	0.66306800	-0.97415500	-0.72463900
O	1.16566700	-0.06822300	1.48262000
H	-2.24103200	1.77638900	0.03789900
O	1.22665400	1.39304700	-0.49561700
H	0.26424600	1.48842900	-0.42754700
C	-2.40737700	-0.08757300	-0.01335400
O	-1.64637000	1.04741600	-0.16670400
O	-3.58002500	0.00998700	0.25779300
O	-1.74461400	-1.17386900	-0.19913100
H	-0.71251800	-1.04456500	-0.38656800

ts14-1
E(RM062X) = -964.803840151 A.U.

S	-1.61841300	0.07241100	-0.09732000
O	-2.72563900	-0.22853600	-0.96881600
O	-0.71187400	1.10961900	-0.59375100
O	-2.01616800	0.28145100	1.37715500
H	-1.26720000	-0.74912000	1.37383700
O	-0.77551900	-1.19298200	0.31556000
H	0.91234800	-1.05789300	-0.12000800
C	2.55911400	-0.00214200	-0.02057300
O	1.87620300	-1.11463100	-0.30279600
O	3.75894300	0.00007800	0.03004800
O	1.84400800	1.09794400	0.20395400
H	0.89513900	1.03774200	-0.06410700

ts14-2
E(RM062X) = -964.830179989 A.U.

S	1.59088100	-0.10344600	0.01747200
O	2.89132900	-0.02244200	-0.60450200
O	0.54943500	-0.68075600	-0.88287000
O	1.52565000	-0.67234400	1.35147500
H	0.21051700	1.43609900	0.44203900
O	1.14075100	1.44987300	0.16762500
H	-1.68793300	1.45052900	-0.90392600
C	-2.47794600	-0.06999700	0.00489500

O	-1.71636000	1.10518000	-0.00610400
O	-3.66848800	-0.01727500	0.13038400
O	-1.76502900	-1.14454700	-0.09396300
H	-0.76729400	-0.95300900	-0.34338300

in14

E(RM062X) = -964.848348869 A.U.

S	1.65018400	-0.08215900	-0.05581900
O	2.80207500	0.40321200	-0.77470300
O	0.41126800	-0.08654100	-0.88028500
O	1.79666800	-1.30559000	0.71413900
H	0.89497700	0.65276800	1.78299000
O	1.36419200	1.07964900	1.05709100
H	-0.97179700	0.89381500	-0.51124300
C	-2.60155600	0.00172000	-0.01607100
O	-1.89721000	1.11147800	-0.25896000
O	-3.75987100	0.03758400	0.29449000
O	-1.93217600	-1.14684900	-0.13734900
H	-0.99634100	-0.98591000	-0.39760300

ts42b

E(RM062X) = -964.798627058 A.U.

S	1.52822500	-0.00420800	0.09129200
O	2.90332300	-0.02454300	-0.32227900
O	0.78075700	-1.13760000	-0.65608600
O	1.21948200	-0.06262300	1.49960900
H	-2.86272100	1.20754400	0.07035200
O	0.89914900	1.30713500	-0.51078900
H	-0.09478800	1.29125000	-0.35513100
C	-2.22698500	-0.16268300	-0.03093200
O	-1.66914600	1.04586100	-0.17267700
O	-3.47221800	0.08517900	0.21206100
O	-1.64776700	-1.24701500	-0.12439200
H	-0.24082600	-1.18652400	-0.39386200

ts2b3b

E(RM062X) = -964.848397358 A.U.

S	-1.79487177	0.75091574	0.00000000
O	-0.41917277	0.70621074	-0.42840400
O	-2.62743377	-0.26576726	-0.76297900
O	-2.06093377	0.66384774	1.42221700
H	-3.80268277	-0.29253426	-0.46526600
O	-2.35342277	2.14966574	-0.51172500
H	-3.33939977	2.13916374	-0.39068500
C	-5.51374777	0.78614674	-0.12952300
O	-4.97190477	-0.35070926	-0.23890700
O	-4.99758877	1.90179874	-0.22680400
O	-6.84983077	0.71851974	0.12314900
H	-7.13703777	1.63403174	0.19611300

ts3b3a

E(RM062X) = -964.833679988 A.U.

S	1.50268000	-0.07607100	0.10158500
O	2.93284400	-0.18127400	-0.06464100
O	0.75116200	-0.98057000	-0.83307900
O	0.97929600	-0.17877800	1.45443100
H	-0.57636200	-1.04466200	-0.56319800
O	1.14181200	1.39781100	-0.42209700
H	0.16403300	1.46010100	-0.40634500
C	-2.14362700	0.05189600	-0.11413100

O	-1.63773800	-1.10721300	-0.32968700
O	-1.62367000	1.13596000	-0.27329100
O	-3.45783200	-0.01950700	0.31279100
H	-3.45577600	-0.04110600	1.27354600

ts2a3a

E(RM062X) = -964.848162833 A.U.

S	1.53955700	-0.06592800	0.07835300
O	2.92209600	-0.12250400	-0.32748600
O	0.69141500	-1.01352100	-0.74840900
O	1.24679500	-0.21863000	1.49058300
H	-0.50684600	-1.03639500	-0.43689900
O	1.02981100	1.37643200	-0.36614000
H	0.04507100	1.39024000	-0.26367100
C	-2.17909900	0.07799600	-0.05275200
O	-1.64975700	-1.08068400	-0.20097400
O	-1.65014500	1.17267200	-0.13625600
O	-3.50983800	0.02851500	0.22596500
H	-3.73956100	-0.90519700	0.26518200

ts2b2a

E(RM062X) = -964.834078851 A.U.

S	1.53128000	-0.00026500	0.10236800
O	2.91916200	-0.00074700	-0.27350300
O	0.85198300	-1.22188700	-0.58605400
O	1.19737500	-0.00037900	1.50891400
H	-0.17914900	-1.19541500	-0.42241300
O	0.85360100	1.22321300	-0.58519600
H	-0.17680000	1.19714600	-0.42219000
C	-2.14993200	0.00016300	-0.09207200
O	-1.62750300	-1.12092500	-0.21354700
O	-1.62753200	1.12107100	-0.21325400
O	-3.52016900	-0.00010600	0.18948900
H	-3.62027200	-0.00040200	1.14435200
