# Supplementary material for: Stabilizing the Exotic Carbonic Acid by Bisulfate Ion
Source: Molecules. 2021 Dec 21;27(1):8. doi: 10.3390/molecules27010008 (PMC8746525; doi:10.3390/molecules27010008)
Supplement: Supplementary file 1 [file molecules-27-00008-s001.zip › Supplementary Materials-Figure S4.pptx]

## Slide 1
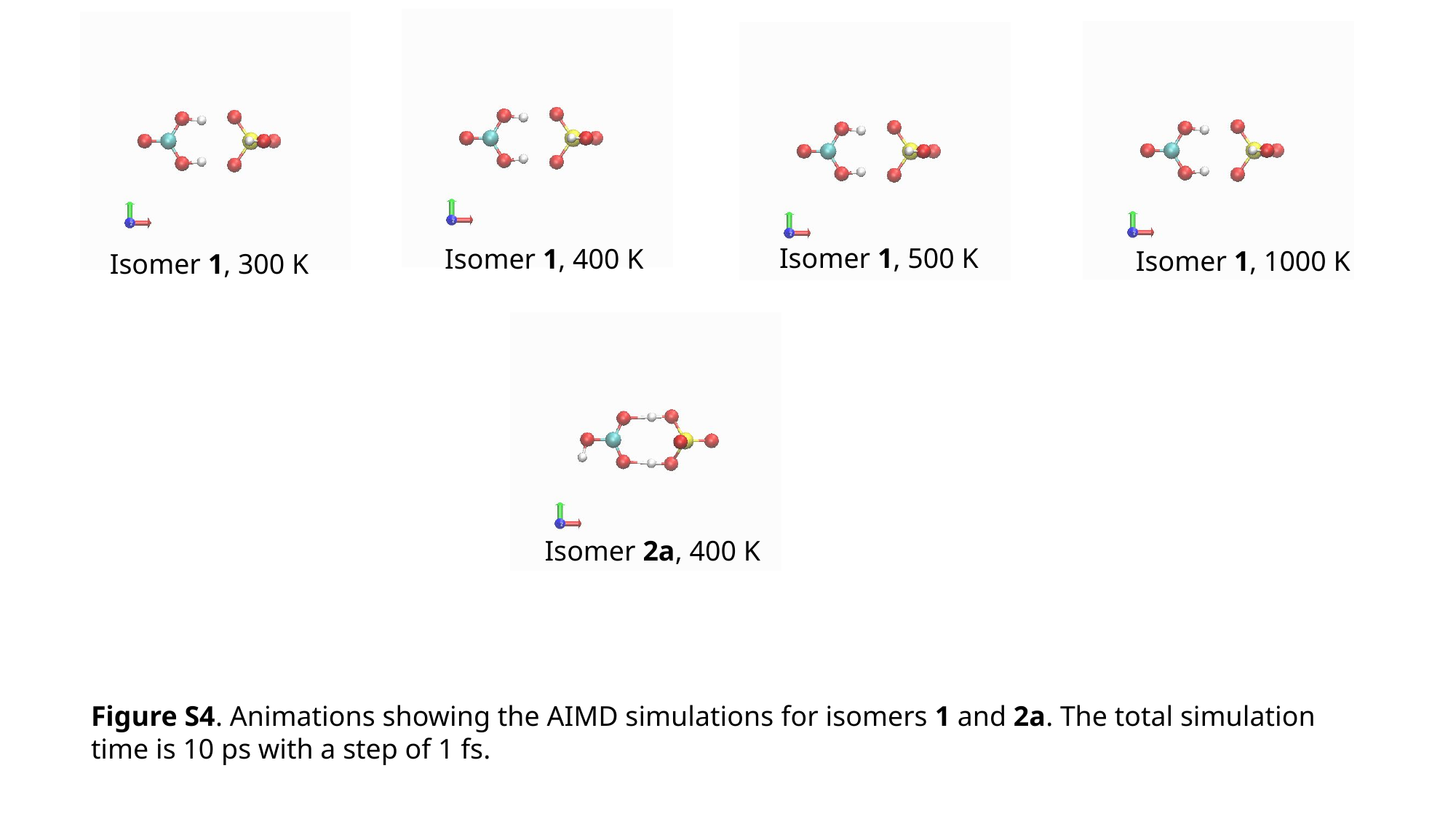

Isomer 1, 500 K
Isomer 1, 400 K
Isomer 1, 1000 K
Isomer 1, 300 K
Isomer 2a, 400 K
Figure S4. Animations showing the AIMD simulations for isomers 1 and 2a. The total simulation time is 10 ps with a step of 1 fs.
